# Item-Specific Factors in IRTree Models: When They Matter and When They Don’t

**DOI:** 10.1007/s11336-023-09916-7

**Published:** 2023-06-16

**Authors:** Thorsten Meiser, Fabiola Reiber

**Affiliations:** Department of Psychology, School of Social Sciences, University of Mannheim, 68131 Mannheim, Germany

**Keywords:** IRTree models, item-specific factors

## Abstract

Lyu et al. (Psychometrika, 2023) demonstrated that item-specific factors can cause spurious effects on the structural parameters of IRTree models for multiple nested response processes per item. Here, we discuss some boundary conditions and argue that person selection effects on item parameters are not unique to item-specific factors and that the effects presented by Lyu et al. (Psychometrika, 2023) may not generalize to the family of IRTree models as a whole. We conclude with the recommendation that IRTree model specification should be guided by theoretical considerations, rather than driven by data, in order to avoid misinterpretations of parameter differences.

Various versions of sequential or treelike IRT models have been proposed over the last years, where the response process to an item is conceptualized as a series of successive steps or logically contingent decisions that together lead to an observed outcome. For example, sequential IRT models for ordinal rating items assume that a positive judgment at some step *h* (i.e., for response categories $\ge h$) leads to a decision on the next step (i.e., for response categories $\ge h+1$), whereas a negative judgment at stage *h* terminates the response process with observed category $h-1$ (Tutz, 1997; Verhelst et al., 1997). In sequential models for repeated attempts of cognitive items, in contrast, a successful response at attempt *h* terminates the process, whereas an incorrect response leads to another attempt $h+1$ until the maximum number of repetitions has been reached (Culpepper, 2014). Other models specify rating judgments related to response intensity or response styles conditional on disagreement versus agreement with the item content, or judgment processes conditional on the non-missingness of an item response (e.g., Böckenholt & Meiser, 2017; Jeon & De Boeck, 2016). Common to these different models is the notion of nested processes, such that the decision at some stage of the response process determines which further processes are involved. Each of the nested processes can be specified in terms of an IRT model equation, leading to the general framework of IRTree models (Böckenholt, 2012; De Boeck & Partchev, 2012), and the probability of the final outcome (e.g., an observed rating response or the number of attempts needed to achieve a correct response) results from the product of the IRT models over the involved processes.

As IRTree models include multiple decisions for each item, like multiple judgments in the choice of a category for a given rating item or multiple responses to the same item in a series of repeated attempts, the assumption of conditional stochastic independence needs to be extended from responses to different items to the multiple decisions within items. Conditional independence of the processes within items is essential inasmuch as the probabilities of observed responses are modeled by multiplication of such processes. Within-item conditional independence is violated, however, if item-specific factors arise due to the unique item content or wording, because such item-specific person effects are not accounted for in IRTree models where the latent traits are considered consistent across items.

## The Role of Neglected Item-Specific Factors in IRTree Models

Lyu et al. (2023) have demonstrated that the neglect of item-specific factors in IRTree models can lead to artifactual effects on the structural IRT parameters of within-item processes. In their conceptual framework of IRTree models with item-specific factors, the authors assume a general trait $\theta$ that is consistent across items, and an item-specific factor $\eta _j$ for each item *j* that is orthogonal to $\theta$ and to all $\eta _{j'}$ for $j' \ne j$. Resembling a bifactor or testlet IRT model (Rijmen, 2010), $\theta$ and $\eta _j$ are further assumed to affect the response processes within item *j* in an additive way. In the IRTree models considered by Lyu et al. (2023), the compensatory nature of $\theta$ and $\eta _j$ leads to a selection of respondents across the conditional within-item processes that is, in turn, mirrored by biases in the item parameters. In a sequential model for ordinal rating responses, for instance, individuals with small values of $\theta + \eta _j$ have a rather low probability of reaching the response stages for higher categories, so that mainly individuals with large values of $\theta +\eta _j$ respond to the later stages of the sequence. As smaller values of $\theta$ can be compensated by larger values of $\eta _j$ and vice versa, the selection process across sequential processes leads to an increasingly negative correlation of $\theta$ and $\eta _j$ and to an attenuation of the effect of $\theta$ on category judgments, as reflected by a decrease in estimated discrimination parameters across the sequential response processes (see Lyu et al., 2023, Figure 1 and Table 1). Similar effects were discussed in the context of IRTree models for repeated item attempts and other multi-process scenarios.

In several simulation studies and reanalyses of empirical data, Lyu et al. (2023) showed that neglected between-item multidimensionality due to item-specific factors can easily be misattributed to changes in the parameters of IRT models over within-item processes, including spurious effects on discrimination and difficulty parameters or changes in the dimensionality of traits across processes. Notwithstanding the authors’ compelling demonstration of potential biases in the results and interpretations of IRTrees due to item-specific factors, in this commentary we want to highlight some limiting conditions for the general claim that item-specific factors play a particular role for selection effects and biases in within-item processes of IRTrees. For this purpose, we first question whether item-specific factors are generally needed to account for person selection effects on IRTree parameters, and second whether biases due to item-specific factors are universal phenomena in IRTree modeling.

## Are Item-Specific Factors Necessary to Induce Selection Effects on Item Parameters?

While Lyu et al. (2023) showed that item-specific factors can contribute to spurious effects on the structural IRT parameters of within-item processes in many cases, the existence of item-specific factors may not be necessary to explain some of their findings. A case in point is the authors’ discussion of response change behavior in a study by Jeon et al. (2017). In the original study, an IRTree model was introduced for assessment designs in which individuals give an initial response to dichotomous performance items and then have the option to revisit their first response. Such designs yield two answers to each item, namely the initial answer and the final answer after a potential change. As each of the two answers can be correct or incorrect, there are four possible outcomes (0, 0), (0, 1), (1, 0) and (1, 1) denoting an incorrect or correct answer at the initial and final stage, respectively.

The IRTree model suggested by Jeon et al. (2017) contains three nested response processes or decision nodes for each item. The first node captures the initial response as incorrect or correct, and the remaining nodes are defined conditional on the first node: The second node represents the final result given that the initial response was incorrect, whereas the third node represents the final result given that the initial response was correct. The probability of a correct answer at each node was modeled in terms of a 2PL model with node-specific item parameters and traits. The IRTree structure and the model specification are summarized in Table 1.

In an empirical application of their IRTree model, Jeon et al. (2017) found that the IRT parameters of the second node showed higher item difficulties than the IRT parameters of the third node.^1^ This observation was interpreted by Lyu et al. (2023) as a potential indication of item-specific factors. More specifically, the authors argued that item-specific factors can affect the correctness of answers as well as the choice to change one’s initial response. Therefore, item-specific factors contribute to selection effects on the side of the persons which can be misattributed to differences in the item parameters between nodes. In the following, we show, however, that selection on the basis of the general trait(s) $\theta$ alone can be sufficient to produce differences in node difficulties and that the assumption of item-specific factors is not necessary or unique in accounting for the observed differences.

Let us first assume that the same trait $\theta$ holds over the three nodes, such that $\theta _1=\theta _2=\theta _3=\theta$ in Table 1. Then, if the items have at least minimum item information for $\theta$, respondents with an incorrect initial answer will have a lower level of $\theta$ on average than respondents with a correct initial answer. Given that the nodes 2 and 3 are defined conditional on the initial response at node 1, the mean of the $\theta$ distribution thus differs between individuals assigned to node 2 and individuals assigned to node 3. Let $\delta$ be the difference in the mean of $\theta$ between nodes 2 and 3. In the original analysis, the expectation of the trait was fixed to zero for each node and thus set to be identical for technical reasons of model implementation (see Jeon et al., 2017, pp. 473f.), so that the actual difference $\delta$ in the person distribution was shifted to differences in the difficulty parameters. This can be seen from the equation $(\theta +\delta )-\beta _{hj} = \theta -(\beta _{hj}-\delta )$. Put differently, in IRT saying that one group of participants has higher proficiency than another group with constant difficulty parameters across groups is tantamount to saying that two groups of participants have identical proficiency but the items are easier for one group than for the other group.

According to this rationale, the differences in difficulty parameters between nodes 2 and 3 obtained by Jeon et al. (2017) can be interpreted as an effect of person selection solely on the basis of the general trait $\theta$ as a function of the initial response, and no item-specific factors are required to explain the result. A similar line of argument holds if different traits apply to the three nodes of the IRTree, as was empirically the case in the original study. It seems plausible to assume that the traits $\theta _2$ and $\theta _3$ at nodes 2 and 3 are composed of the domain ability $\theta _1$ measured at node 1 and some additional person characteristics pertaining to an individual’s metacognitive uncertainty or motivation that affect answer change (Jeon et al., 2017, p. 471). The traits $\theta _2$ and $\theta _3$ can then be written as $\theta _2 = \theta _1 + \theta _2^*$ and $\theta _3 = \theta _1 + \theta _3^*$, where $\theta _2^*$ and $\theta _3^*$ reflect the additional node-specific person effects. It can easily be seen that mean differences in domain ability $\theta _1$ between participants assigned to node 2 and node 3 are mirrored by differences in the difficulty parameters if the trait distribution is centered for each node.

As the example of response change illustrates, item-specific factors are not the only source of person selection effects that may be mirrored in item parameters, and they are not unique in explaining changes in the structural item parameters across IRTree nodes.

## Do Item-Specific Factors Induce Selection Effects on IRTree Parameters in General?

In their conceptual analysis of item-specific factors, Lyu et al. (2023) mainly focused on IRTree models in which the nested decision processes are unidirectional. Sequential models for rating responses, for instance, presume that a positive judgment at one stage of the response process transfers to a decision on a subset of higher response categories, so that subsequent decisions are ordered from lower to higher categories (i.e., “linear tree models,” De Boeck & Partchev, 2012). Alternative IRTree models for rating responses decompose the response process in a non-directional way, however, and distinguish between a judgment of (dis)agreement with the item content and symmetrical judgments of response intensity (e.g., Böckenholt, 2012, 2017; Jeon & De Boeck, 2016; Meiser et al., 2019; Merhof & Meiser, 2023). For example, Table 2 summarizes an IRTree model for 6-point rating responses with symmetrical intensity decisions, where node 1 reflects the (dis)agree judgment and nodes 2 and 3 reflect gradual judgments of the strength of disagreement or agreement alike. The traits $\theta _2$ and $\theta _3$ can be conceived of as distinct response styles of non-midscale and extreme responding, as one dimension of intensity $\xi =\theta _2=\theta _3$, or as compounds of response styles and the target trait of measurement (see Meiser et al., 2019, for details).

Considering the conceptual model of item-specific factors $\eta _j$ that affect the degree of item agreement monotonically together with a target trait $\theta$ (Lyu et al., 2023), the symmetrical definition of the response processes at nodes 2 and 3 in Table 2 prohibit directional selection effects along $\theta + \eta _j$ over increasing or decreasing categories. Item-specific factors $\eta _j$ would thus not be expected to cause systematic effects on model parameters in the IRTree nodes 2 and 3 that are coded orthogonal to $\eta _j$. If, on the other hand, one assumes item-specific factors operating in the direction of response intensity instead of monotonically increasing levels of agreement, one would predict selection effects toward more extreme categories of disagreement and agreement, respectively. As a consequence, the impact of the trait $\theta _3$ at node 3 should be attenuated relative to that of trait $\theta _2$ at node 2. This implication is the opposite of empirical findings, however, that were obtained in an empirical application showing that the latent trait variance of $\theta _3$ was larger than the latent trait variance of $\theta _2$ (Meiser et al., 2019, p. 513).^2^

Therefore, effects of item-specific factors on structural parameters are not universal in IRTree models with nested response processes per se, and further research should provide insight into the conditions that make IRTree models susceptible to spurious effects of item-specific factors.

## Conclusions

Lyu et al. (2023) have pointed at an important source of model misspecification in IRT models with multiple nested decisions per item, namely the existence of neglected item-specific factors that violate conditional independence of within-item processes. Like any other model misspecification, ignored systematic variance of item-specific factors can cause biases in estimated model parameters, and Lyu et al. (2023) presented convincing evidence that such biases can include misattributions of item-specific person effects to differences in the structural parameters of nested response processes. In this commentary, we have delineated that some results discussed by Lyu et al. (2023) might also be explained without assuming item-specific factors and that biases outlined by the authors do not generalize to all IRTree models, so that effects of item-specific factors on structural parameters should not be regarded as unique and universal. Nevertheless, since item-specific factors are likely to prevail in many empirical scenarios where multiple processes relate to the same item, the work by Lyu et al. (2023) highlights a caveat to the validity of IRTree models that researchers have to address. One crucial recommendation that follows from the analysis of Lyu et al. (2023) is that researchers should specify IRTree models in a theoretically motivated and parsimonious way, rather than driven by empirical data. By keeping theoretically meaningful parameter constraints, one can avoid the overinterpretation of parameter differences and the misinterpretation of spurious effects due to item-specific factors.

**Table 1:** IRTree model of response change behavior proposed by Jeon et al. (2017).

	(0,0)	(0,1)	(1,0)	(1,1)	
Node 1	0	0	1	1	$p(Y_{1pj}=1)=\frac{\displaystyle \exp \left( \alpha _{1j}(\theta _{1p} - \beta _{1j}) \right) }{\displaystyle 1 + \exp \left( \alpha _{1j}(\theta _{1p} - \beta _{1j}) \right) }$
Node 2	0	1	–	–	$p(Y_{2pj}=1)=\frac{\displaystyle \exp \left( \alpha _{2j}(\theta _{2p} - \beta _{2j}) \right) }{\displaystyle 1 + \exp \left( \alpha _{2j}(\theta _{2p} - \beta _{2j}) \right) }$
Node 3	–	–	0	1	$p(Y_{3pj}=1)=\frac{\displaystyle \exp \left( \alpha _{3j}(\theta _{3p} - \beta _{3j}) \right) }{\displaystyle 1 + \exp \left( \alpha _{3j}(\theta _{3p} - \beta _{3j}) \right) }$

The symbol “–” denotes missing values by design that follow from the nested definition of nodes.

$\alpha _{hj}$: discrimination parameter for node *h* in item *j*; $\beta _{hj}$: difficulty parameter for node *h* in item *j*; $\theta _{hp}$: value of person *p* on trait *h*; $h=1,2,3$.

**Table 2:** IRTree model of (dis)agreement and response intensity for 6-point rating items.

	Disagree			Agree			
	$\longleftarrow$			$\longrightarrow$			
	1	2	3	4	5	6	
Node 1	0	0	0	1	1	1	$p(Y_{1pj}=1)=\frac{\displaystyle \exp \left( \theta _{1p} - \beta _{1j}\right) }{\displaystyle 1 + \exp \left( \theta _{1p} - \beta _{1j} \right) }$
Node 2	1	1	0	0	1	1	$p(Y_{2pj}=1)=\frac{\displaystyle \exp \left( \theta _{2p} - \beta _{2j}\right) }{\displaystyle 1 + \exp \left( \theta _{2p} - \beta _{2j} \right) }$
Node 3	1	0	–	–	0	1	$p(Y_{3pj}=1)=\frac{\displaystyle \exp \left( \theta _{3p} - \beta _{3j}\right) }{\displaystyle 1 + \exp \left( \theta _{3p} - \beta _{3j} \right) }$

The symbol “–” denotes missing values by design that follow from the nested definition of nodes.

$\beta _{hj}$: difficulty parameter for node *h* in item *j*; $\theta _{hp}$: value of person *p* on trait *h*; $h=1,2,3$.

## Footnotes

1 Because Jeon et al. (2017) used a different notation, the item difficulty parameters $\beta _{hj}$ according to Table 1 can be computed from the original discrimination and intercept parameters $\alpha _{hj}$ and $\gamma _{hj}$ (Jeon et al., 2017, Figure 3) as $\beta _{hj} = - \frac{\displaystyle \gamma _{hj}}{\displaystyle \alpha _{hj}}$ for node $h \in \{1,2,3\}$ and item *j*.

2 Because the discrimination parameters were set to 1.0 in the analysis of Meiser et al. (2019), as illustrated in Table 2 here, the strength of the trait effect was reflected in the estimated latent variances rather than in estimated discrimination parameters.

## Declaration

### Data Availability

This manuscript does not report any original data that could possibly be made available to other researchers and readers.

## References

[CR1] Böckenholt U (2012). Modeling multiple response processes in judgment and choice. Psychological Methods.

[CR2] Böckenholt U (2017). Measuring response styles in Likert items. Psychological Methods.

[CR3] Böckenholt U, Meiser T (2017). Response style analysis with threshold and multi-process IRT models: A review and tutorial. British Journal of Mathematical and Statistical Psychology.

[CR4] Culpepper SA (2014). If at first you don’t succeed, try, try again: Applications of sequential IRT models to cognitive assessments. Applied Psychological Measurement.

[CR5] De Boeck P, Partchev I (2012). IRTrees: Tree-based item response models of the GLMM family. Journal of Statistical Software.

[CR6] Jeon M, De Boeck P (2016). A general item response tree model for psychological assessments. Behavior Research Methods.

[CR7] Jeon M, De Boeck P, van der Linden W (2017). Modeling answer change behavior: An application of a generalized item response tree model. Journal of Educational and Behavioral Statistics.

[CR8] Lyu W, Bolt DM, Westby S (2023). Exploring the effects of item-specific factors in sequential and IRTree models. Psychometrika.

[CR9] Meiser T, Plieninger H, Henninger M (2019). IRTree models with ordinal and multidimensional decision nodes for response styles and trait-based rating responses. British Journal of Mathematical and Statistical Psychology.

[CR10] Merhof V, Meiser T (2023). Dynamic response strategies: Accounting for response process heterogeneity in IRTree decision nodes. Psychometrika.

[CR11] Rijmen F (2010). Formal relations and an empirical comparison among the bi-factor, the testlet, and the second-order multidimensional IRT model. Journal of Educational Measurement.

[CR12] Tutz G, van der Linden WJ, Hambleton RK (1997). Sequential models for ordered responses. Handbook of modern item response theory.

[CR13] Verhelst, N. D., Glass, C. A. W., & de Vries, H. H. (1997). A steps model to analyze partial credit. In W. J. van der Linden & R. K. Hambleton (Eds.), *Handbook of modern item response theory* (pp. 123–138). Springer.

